# Severe hypertriglyceridemia in Norway: prevalence, clinical and genetic characteristics

**DOI:** 10.1186/s12944-017-0511-9

**Published:** 2017-06-12

**Authors:** Kjetil Retterstøl, Ingunn Narverud, Randi Selmer, Knut E. Berge, Ingvild V. Osnes, Stine M. Ulven, Bente Halvorsen, Pål Aukrust, Kirsten B. Holven, Per O. Iversen

**Affiliations:** 1 Department of Nutrition, Institute of Basic Medical Sciences, University of Oslo, P.O. Box 1046, Blindern, 0317 Oslo, Norway; 2 Lipid Clinic, Oslo University Hospital, Oslo, Norway; 3 National Advisory Unit on Familial Hypercholesterolemia, Oslo University Hospital, Oslo, Norway; 4 The Norwegian Institute of Public Health, Oslo, Norway; 5 Unit for Cardiac and Cardiovascular Genetics, Department of Medical Genetics, Oslo University Hospital, Oslo, Norway; 6 Research Institute of Internal Medicine, Oslo University Hospital, Oslo, Norway; 7 K.G. Jebsen Inflammatory Research Center, University of Oslo, Oslo, Norway; 8 Section of Clinical Immunology and Infectious Diseases, Oslo University Hospital, Oslo, Norway; 9 Department of Haematology, Oslo University Hospital, Oslo, Norway

**Keywords:** Co-morbidity, Genetics, Pancreatitis, Prevalence, Triglycerides

## Abstract

**Background:**

There is a lack of comprehensive patient-datasets regarding prevalence of severe hypertriglyceridemia (sHTG; triglycerides ≥10 mmol/L), frequency of co-morbidities, gene mutations, and gene characterization in sHTG. Using large surveys combined with detailed analysis of sub-cohorts of sHTG patients, we here sought to address these issues.

**Methods:**

We used data from several large Norwegian surveys that included 681,990 subjects, to estimate the prevalence. Sixty-five sHTG patients were investigated to obtain clinical profiles and candidate disease genes. We obtained peripheral blood mononuclear cells (PBMC) from six male patients and nine healthy controls and examined expression of mRNAs involved in lipid metabolism.

**Results:**

The prevalence of sHTG was 0.13 (95% CI 0.12-0.14)%, and highest in men aged 40-49 years and in women 60-69 years. Among the 65 sHTG patients, a possible genetic cause was found in four and 11 had experienced acute pancreatitis. The mRNA expression levels of *carnitine palmitoyltransferase (CPT)-1A, CPT2*, and *hormone-sensitive lipase*, were significantly higher in patients compared to controls, whereas those of *ATP-binding cassette, sub-family G, member 1* were significantly lower.

**Conclusions:**

In Norway, sHTG is present in 0.1%, carries considerable co-morbidity and is associated with an imbalance of genes involved in lipid metabolism, all potentially contributing to increased cardiovascular morbidity in sHTG.

**Electronic supplementary material:**

The online version of this article (doi:10.1186/s12944-017-0511-9) contains supplementary material, which is available to authorized users.

## Background

Severe hypertriglyceridemia (sHTG) can be defined as a non-fasting serum concentration of triglycerides (TG) ≥10 mmol/L. The frequency of risk factors for sHTG (e.g. obesity, diabetes and high alcohol consumption) has increased in the last decades, calling for increased awareness of sHTG, and at present the prevalence in the general population is still uncertain.

There are upcoming possible therapies for sHTG such as apolipoprotein CIII inhibitors or lopitapide [1]. In addition, a gene therapy product for lipoprotein lipase deficiency has been approved by the European Medicines Agency [2], and monoclonal antibody-based therapy has also been applied [3]. To utilize these novel therapeutic modalities there is a need for more data on the prevalence and consequences of sHTG.

In the present study we therefore aimed (i) to describe the prevalence of sHTG in Norway by surveying several large cohorts; (ii) to present detailed clinical data of patients with sHTG; and (iii) to examine the expression of lipid-related genes in peripheral blood mononuclear cells of patients with hypertriglyceridemia.

## Methods

The study protocol was approved by the Regional Committee for Medical Health Research Ethics (#S-08337a/S-07455a) and by the Norwegian Data Inspectorate (#08/3914). A written informed consent was obtained from each patient included in the study and the study protocol conformed to the ethical guidelines of the 1975 Declaration of Helsinki.

### Collection of data from three surveys to estimate the prevalence of sHTG

The current data on prevalence originates from (i) the COhorts of NORway (*CONOR*; *n* = 172,956); (ii) the *3 Counties Study* (*n* = 92,080); and (iii) the *40 Years Survey* (*n* = 416,954). The data from these three surveys have in the present study been merged (*n* = 681,990) to generate data of the Norwegian population.

*CONOR* covers 10 population-based studies during 1994-2003 from different parts of Norway, both urban and rural regions. Some studies invited all residents above a specific age (for example all above 19 years in the HUNT 2 study), whereas others invited all subjects in selected age groups (for example all 30-, 40-, 45-, 60 and 75 years in the OPPHED- and TROFINN studies). The overall participation rate was 60% and varied between the studies. The participants (aged 20-103 years) filled in a questionnaire covering physical activity, history of previous cardiovascular disease (CVD), diabetes and use of medicine. A non-fasting blood sample was collected. Body mass index (BMI) and systolic blood pressure were also measured. For subjects who participated more than once we used the last measured TG values. Details of the research tools and the measurements of blood chemistry have been described elsewhere [4].

In the *3 Counties Study* (covering the counties Oppland in Eastern part of Norway; Sogn og Fjordane in Western part of Norway; and Finnmark in Northern part of Norway; during 1974-1988) all residents aged 35-49 years at the study start in each county were invited to the first screening. The study had three visits in each county. The same birth cohorts and in some counties broader age groups, were invited to the second and third screening in each county. Altogether about 93,000 men and women participated in at least one screening. The participation rate varied from 78% to 90%. The participants were screened for CVD risk factors and a non-fasting blood sample was collected. We have included the last measured TG value for each participant in the present study. Details of the research tools and the measurements of blood chemistry have been described elsewhere [5, 6].

The *40 Years Survey* was conducted from 1985 to 1999 and consisted of 429,245 participants from 18 counties. All inhabitants aged 40-42 years were invited to the survey whereas in some counties and municipalities broader age groups were included. The participation rate varied from 69% in Nord-Trøndelag county in 1992 to 46% in Østfold- and Aust-Agder counties in 1999. A non-fasting blood sample was collected. A few subjects participated more than once, and for them the last TG value was used in the present study. Details of the research tools and the measurements of blood chemistry have been described elsewhere [7].

From each study, we included all participants who had obtained measurements of serum triglycerides and who had answered the questionnaires.

### Collection of data from patients with sHTG for detailed analyses

All individuals >18 years treated for sHTG at the Lipid Clinic from January 1st 2002 to December 31st 2007 were identified by searching the medical records for the diagnoses (ICD-10 classification): pure hypertriglyceridemia (E78.1), mixed hyperlipidemia (E78.2) and hyperchylomicronemia (E78.3). To ensure that the hypertriglyceridemia was not transient, only patients with long-lasting sHTG was included, i.e. they were diagnosed with sHTG both at the time of referral and at two consecutive time points 2-6 months apart. One hundred and twelve patients fulfilled the inclusion criteria and were invited by letter. Sixty-five (58%) patients returned a signed informed consent and were included in the study and came for a baseline visit. Inclusion and exclusion criteria are listed in the Additional file 1: Table S1. Data regarding age, diagnoses, clinical findings, laboratory measures, and use of medication were collected from their medical records. All laboratory values were collected after at least 12 h of fasting and analysed at the laboratory at Oslo University Hospital, using standard procedures. Most patients were controlled after 0.5, 1, 2 and 3 years (± 3 months) after the baseline visit. The genes *lipoprotein lipase* (*LPL*), *apolipoprotein C-II* (*APOC2*), *apolipoprotein* A-V (APOA5)*, glycosylphosphatidylinositol-anchored high density lipoprotein-binding protein 1* (*GPIHBP1*) and *lipase maturation factor 1* (*LMF1*) were sequenced at the Unit for Cardiac and Cardiovascular Genetics, Department of Medical Genetics, Oslo University Hospital, and apolipoprotein E was genotyped. Information regarding diet and lifestyle were collected from the validated food questionnaire SmartDiet [8].

### RNA expression of lipid-related genes in peripheral blood mononuclear cells

Six male patients with hypertriglyceridemia and nine male control subjects were recruited from employees of the Lipid Clinic and the University of Oslo, respectively, to this sub-study.

We analyzed a range of lipid-related genes (Additional file 1: Table S2) in peripheral blood mononuclear cells (PBMC) obtained from heparinized blood by gradient centrifugation in Isopaque-Ficoll (Lymphoprep, Nycomed, Oslo, Norway). The assay-numbers (hs-numbers) of the specific genes chosen for the analysis are included (Additional file 1: Table S2). PBMC pellets for mRNA expression analyses were immediately frozen and stored at −80 °C. Total RNA was isolated from all PBMC samples using RNeasy mini kit (Qiagen, Hilden, Germany) with lysis buffer containing β-mercaptoethanol, and RNase-Free DNase (Qiagen) and stored at −80 °C. RNA quantity and -quality measurements were performed using the ND 1000 Spectrophotometer (Saveen Werner, Carlson Circle Tampa, FL) and Agilent Bioanalyser (Agilent Technologies, Santa Clara, CA), respectively. Four hundred ng RNA from all samples were reverse-transcribed using High Capacity RNA-to-cDNA Kit (Applied Biosystems, Foster City, CA). Quantitative real-time polymerase chain reaction (RT-qPCR) was performed using Custom TaqMan Array micro Fluidic cards (Applied Biosystems). The endogenous control genes *glucuronidase β* (*GUSβ*) and *TATA box binding protein* (*TBP*) were used for normalization. The relative mRNA level for each transcript was calculated by the ΔΔ cycle threshold method [9].

### Statistical analyses

The patient data were analysed with the Statistical Package for the Social Sciences (SPSS), version 16.0. Results from non-parametric statistical analyses are reported as median and 95% confidence interval (95% CI). Results from variables that showed a normal distribution are reported as mean and standard deviation (SD). For comparisons of two groups of individuals, the Mann-Whitney *U* test was used**.** Significance was assumed for *p* < 0.05. The prevalence data were analyzed with SPSS version 19.

## Results

### Prevalence of hypertriglyceridemia

The prevalence of subjects with non-fasting serum TG concentrations ≥10 mmol/L, i.e. sHTG, was 0.13 (95% CI 0.12-0.14) % based on pooled data from the three different surveys, while 2.0% of the participants had moderately increased TG levels (i.e. between 5.0 and 9.9 mmol/L) (Table 1). Furthermore, about 28% had TG levels above 2 mmol/L, a cut-off value often used for diagnosing hypertriglyceridemia [10]. Notably these pooled estimates stem from different surveys with somewhat different inclusion criteria, age distributions and time periods.

**Table 1:** Distribution of non-fasting serum triglycerides in Norwegian population surveys

		Triglyceride concentration (mmol/L)			
Survey	Participants^a^	< 2.0	2.0 – 4.9	5.0 – 9.9	≥ 10.0
	*n*	*n* (%)	*n* (%)	*n* (%)	*n* (%)
*CONOR*	172,956	125,455 (72.54)	44,408 (25.68)	2919 (1.69)	174 (0.10)
*3 Counties Study*	92,080	62,928 (68.34)	27,071 (29.40)	1955 (2.12)	126 (0.14)
*40 Years Survey*	416,954	299,080 (71.73)	108,615 (26.05)	8695 (2.09)	562 (0.13)
Total	681,990	487,463 (71.48)	180,094 (26.41)	13,569 (1.99)	862 (0.13)

^a^For persons who participated more than once, the last triglyceride value was used in the present study

### Hypertriglyceridemia in the CONOR survey- relation to other risk factors

The *CONOR* survey provided further age- and gender specific data of the included participants. The prevalence of sHTG was significantly higher in men than in women for the three age groups between 30 and 59 years (Table 2). Moreover, the prevalence peaked in the age group 40-49 years for men and in the age group 60-69 years for women. We next examined whether various known risk factors for cardiovascular disease were associated with elevated TG values (Table 3). A non-fasting serum concentration of glucose ≥11 mmol/L was present in 15.4% of those with sHTG, compared to only 0.7% of subjects with non-fasting TG < 2 mmol/L (*p* < 0.05) indicating that high serum glucose was more than 20 times more prevalent in sHTG subjects. The prevalence of systolic blood pressure above 160 mmHg was similar in those with sHTG (13.9%) and those with non-fasting TG < 2 mmol/L (10.2%) (*p* > 0.05). Furthermore, among the sHTG subjects, a BMI ≥ 30 kg/m^2^ was present in 41.6% in comparison to 10.8% of subjects with non-fasting TG < 2 mmol/L (*p* < 0.05). When asked if they had ever experienced a myocardial infarction (MI), 2.7% of the subjects with TG < 2 mmol/L answered yes compared to 5.8% of those with sHTG (*p* < 0.05). Only 6% of sHTG subjects reported to be physical active ≥3 h/week compared to 13.7% among those with TG < 2 mmol/L (*p* < 0.05). Daily smoking was reported by 29.9% of subjects with TG levels <2 mmol/L and in 48.6% of those with sHTG (*p* < 0.05).

**Table 2:** Prevalence of severe hypertriglyceridemia in the *CONOR* survey

	Men			Women		
Age group (years)	sHTG	*n*	Prevalence^a^ (95% CI)	sHTG	*n*	Prevalence^a^ (95% CI)
0 – 29	2	5274	38 (5 – 137)	1	6458	15 (0 – 86)
30 – 39	22	12,921	170 (107 – 258)	1	15,126	7 (0 – 37)
40 – 49	73	28,156	259 (203 – 326)	16	32,376	49 (28 – 80)
50 – 59	21	9847	213 (132 – 326)	3	9687	31 (6 – 90)
60 – 69	12	12,278	98 (51 – 171)	10	10,890	92 (44 – 169)
70 – 79	5	13,925	36 (12 – 84)	6	11,879	51 (19 – 110)
80 – 89	0	1604	ND	2	2535	79 (10 – 285)

^a^The prevalence data are expressed per 100,000 subjects. *sHTG* severe hypertriglyceridemia, *CI* confidence interval, *ND* not detected

**Table 3:** Distribution of biochemistry, clinical and lifestyle characteristics in relation to serum triglycerides in the *CONOR* survey

	Non-fasting trigycerides (mmol/L)									
	< 2.0		2.0-4.9		5.0-9.9		≥ 10		Total	
	*n*	%	*n*	%	*n*	%	*n*	%	*n*	%
Non-fasting serum glucose (mmol/L)										
< 7.0	103,889	94.5	35,527	88.0	2134	80.2	119	73.5	141,669	92.5
7.0-8.9	4432	4.0	3065	7.6	262	9.8	16	9.9	7775	5.1
9.0-10.9	855	0.8	759	1.9	95	3.6	2	1.2	1711	1.1
≥ 11.0	734	0.7	1006	2.5	170	6.4	25	15.4	1935	1.3
Total	109,910	100.0	40,357	100.0	2661	100.0	162	100.0	153,090	100.0
Systolic blood pressure (mmHg)										
< 160	112,395	89.8	36,850	83.3	2376	81.5	149	86.1	151,770	88.0
≥ 160	12,715	10.2	7383	16.7	538	18.5	24	13.9	20,660	12.0
Total	125,110	100.0	44,233	100.0	2914	100.0	173	100.0	172,430	100.0
Body mass index (kg/m^2^)										
< 20	6120	4.9	350	0.8	5	0.2	0	0.0	6475	3.8
20.0-24.9	58,443	46.8	9791	22.2	339	11.7	16	9.2	68,589	39.9
25.0-29.9	46,690	37.4	22,380	50.8	1464	50.5	85	49.1	70,619	41.1
≥ 30.0	13,501	10.8	11,506	26.1	1092	37.7	72	41.6	26,171	15.2
Total	124,754	100.0	44,027	100.0	2900	100.0	173	100.0	171,854	100.0
Have had myocardial infarction										
Yes	3371	2.7	2193	5.0	146	5.1	10	5.8	5720	3.4
No	120,420	97.3	41,489	95.0	2718	94.9	163	94.2	164,790	96.6
Total	123,791	100.0	43,682	100.0	2864	100.0	173	100.0	170,510	100.0
Hard physical activity during one week										
No	38,733	36.5	15,432	43.0	1089	46.0	58	38.4	55,312	38.3
< 1 h	26,506	25.0	9176	25.6	597	25.2	50	33.1	36,329	25.1
1 - 2 h	26,371	24.8	7328	20.4	449	19.0	34	22.5	34,182	23.6
≥ 3 h	14,575	13.7	3917	10.9	233	9.8	9	6.0	18,734	13.0
Total	106,185	100.0	35,853	100.0	2368	100.0	151	100.0	144,557	100.0
Daily smoking										
No	87,015	70.1	30,394	69.4	1862	64.7	89	51.4	119,360	69.8
Yes	37,129	29.9	13,380	30.6	1016	35.3	84	48.6	51,609	30.2
Total	124,144	100.0	43,774	100.0	2878	100.0	173	100.0	170,969	100.0

### Clinical, genetic and biochemical profiles of patients with severe hypertriglyceridemia

To give a more in-depth characterization of patients with sHTG, 65 subjects who agreed to participate (51 men and 14 women) were further characterized by clinical, genetic and biochemical methods. Among these 65 patients, 28 were diagnosed with a primary hypertriglyceridemia whereas the remaining 37 had a secondary hypertriglyceridemia.

Among the men, the mean (± SD) age was 45.6 ± 9.1 years and the mean BMI was 29.2 ± 4.0 kg/m^2^. Among the women, the mean age was 46.7 ± 6.4 years and the mean BMI was 29.4 ± 4.6 kg/m^2^. In four of the 28 patients with primary hypertriglyceridemia a possible genetic cause of the sHTG was found: Homozygosity for a deletion comprising exons 3 and 4 in *GPIHBP1* was found in two women who both had been hospitalized for pancreatitis several times [11]. Another patient suffered from type III hyperlipidemia due to apolipoprotein E2/E2 homozygosity. One patient was heterozygous for the two non-synonymous SNPs: c.106G > A (rs1801177) and c.998G > A (rs144466625) (Ref. seq.: NM _000237.2) in the *LPL* gene. We do not know whether these SNPs reside on different alleles. No mutations were found in *LMF1*, *APOC2* or *APOA5*.

The values of the biochemical characteristics of the 65 patients with sHTG were within the standard reference limits except for high values of total cholesterol and TG (Additional file 1: Table S3). The median and maximum fasting serum TG values at the first visit were 12.3 and 57.3 mmol/L, respectively. Forty-eight percent of the patients smoked daily, 50% were overweight (BMI > 25 kg/m^2^), 36% were obese (BMI > 30 kg/m^2^), and 43% were physically inactive (< 30 min of physical activity maximally once per week).

**Fig. 1:**
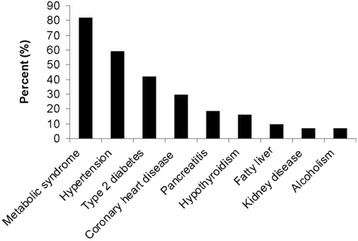
Frequency distribution of comorbidities in the 65 patients with severe hypertriglyceridemia

Metabolic syndrome, hypertension, high alcohol intake, diabetes mellitus type 2 and other conditions known to be associated with sHTG were observed in 61 of the 65 (94%) patients (Fig. 1). In total, 11 (17%) patients had experienced pancreatitis (4 with one episode and 7 with recurrent episodes) and all had co-factors known to increase the risk for pancreatitis (alcohol abuse, *n* = 4 and diabetes type 2, *n* = 7). At the first measurement after initiation of treatment, patients who had experienced acute pancreatitis (mean TG 22.1 mmol/L) had significantly higher TG values than patients without pancreatitis (mean TG 8.2 mmol/L). Eleven of the patients with sHTG had a medical history of coronary artery disease and eight other patients experienced a coronary event during the study period.

All 65 patients had received lifestyle advice and diet therapy from a clinical nutritionist at least once, whereas 33 used one or several lipid-lowering medications when referred to the Lipid Clinic, increasing to 43 at the last consultation (Additional file 1: Table S4). None used medication known to increase TG. Among the 65 patients, 10 had reached a serum TG concentration within the laboratory reference value of 2.6 mmol/L or lower one year after the first visit at the Lipid Clinic. Due to the small number of this subset compared to the much higher number in the larger surveys, we cannot readily make comparisons between them.

### PBMC expression of lipid-related genes

In the subgroup of six male hypertriglyceridemic patients and 9 male control subjects, mRNA levels of relevant genes involved in lipid metabolism were analysed in PBMC since previous studies have shown that dyslipidemic alterations are reflected in the PBMC transcriptome [12, 13]. Their characteristics are displayed in the Additional file 1: Table S5. None of them had cardiovascular disease. These patients had significantly higher levels of total cholesterol, TG and glucose, and lower levels of apolipoprotein A1 compared to the controls (Table 4).

**Table 4:** Characteristics of the male sub-sample with gene expression profiling and cholesterol efflux measurements compared with male control subjects

	Hypertriglyceridemia	Controls	*p*-values
	*n* = 6	*n* = 9	
Age (years)	44 (32-50)	52 (25-60)	0.41
Total cholesterol (mmol/L)	8.9 (5.2-14.6)	4.5 (3.7-5.6)	0.004
LDL cholesterol (mmol/L)	2.3 (1.9-3.8)	2.9 (2.0-4.1)	0.41
HDL cholesterol (mmol/L)	1.4 (0.8-2.4)	1.5 (1.1-2.0)	0.63
Triglycerides (mmol/L)	17.3 (5.1-58)	0.7 (0.4-1.5)	0.001
Apolipoprotein A1 (mg/L)	1.1 (1.1-1.4)	1.5 (1.1-1.7)	0.019
Apolipoprotein B (mg/L)	0.8 (0.7-1.1)	0.8 (0.6-1.1)	0.58
Free fatty acids (nmol/L)	593 (372-798)	387 (172-681)	0.090
Hs-CRP (mg/L)	2.4 (0.7-20)	1.1 (0.7-2.8)	0.20
Glucose (mmol/L)	10.1 (5.5-14.7)	5.0 (4.4-5.4)	0.001

Data are presented as median (range)

*Hs-CRP* high sensitivity C-reactive protein

The mRNA expression levels of *CPT-1A* and *CPT-2,* encoding mitochondrial enzymes involved in β-oxidation of fatty acids, were significantly higher in the patients compared to the controls (Additional file 2: Figure S1). The mRNA expression of *LIPE,* encoding the hormone sensitive lipase that hydrolyzes stored TG to free fatty acids, was also significantly elevated among the patients compared to the controls. In contrast, the mRNA expression of *ABCG1*, encoding a protein contributing to cholesterol efflux capacity, was significantly lower in the HTG patients compared to the controls.

## Discussion

In the present study we show that the prevalence of sHTG in Norway is about 0.1%, and associated with a number of serious co-morbidities such as CVD and pancreatitis. The high prevalence of elevated non-fasting glucose concentrations, obesity and low physical activity suggest a disturbed energy metabolism in these subjects. Moreover, in a subgroup of patients, we found that the hypertriglyceridemic phenotype was associated with changes in gene expression of key players in lipid metabolism, potentially contributing to the high prevalence of CVD in these patients.

There are few nation-wide data on the prevalence of hypertriglyceridemia, and there is no established definition of sHTG. Reportedly 1.7% of adults in the United States 2001-2006 had elevated fasting TG values ranging from 5.6 to 22.6 mmol/L [14], whereas we found that 2% of the subjects had moderately elevated non-fasting TG levels (5-10 mmol/L) in Norway, similar to the 1.9% recently reported in Denmark [10]. In addition, about 25% of US adults had hypertriglyceridemia (TG values >2 mmol/L), i.e. about the same estimate as we report here (28%). In comparison, among otherwise healthy adult Iranians, nearly 10% had hypertriglyceridemia in 2013 [15], whereas the corresponding percentage of hypertriglyceridemia in Västerbotten County, Sweden in 2008-2010 was 15.5 and 31.9 among women and men, respectively [16]. In the present study 20% of the women and 36% of the men had TG values >2 mmol/L, based on the data from the CONOR survey. Collectively, our Norwegian data are quite similar to the quoted estimates of the prevalence of hypertriglyceridemia obtained in populations from the US [14], Iran [15] and Sweden [16]. Notably, these populations differ in relation to life-styles and risks of CVD. Moreover, the data from the CONOR-survey consistently showed that sHTG level was associated with a number of risk factors for CVD, diabetes and the metabolic syndrome, as evidenced by elevated glucose, blood pressure, BMI as well as low physical activity [17]. If these risk factors represent potential causes or consequences of sHTG or if they are associated phenomena reflecting common pathogenic mechanisms such as those leading to metabolic syndromes, are at present not known.

The 28 patients with primary hypertriglyceridemia who were included in the sHTG sub-study had persistent sHTG for several months, but in only four of them was a possible genetic cause found, demonstrating that even in a long lasting sHTG a genetic cause is usually not found among currently known predisposing genes. Unfortunately we do not have data regarding the prevalence of heterozygous mutations in the 65 patients. The fasting TG values were reduced by nearly 60% after treatment, a reduction that seemed to persist for years. In line with others we observed that sHTG was associated with several serious co-morbidities [18]. Notably, 19 of the 65 patients (29%) had established coronary heart disease and eight of these patients experienced a coronary event during the study period, suggesting that they were at significant risk even after treatment with marked decrease in TG levels. Without a control group we cannot exclude the possibility that the fall in TG levels is the natural evolution of the disease (sHTG). However, the relatively rapid effect of treatment observed after 6 months, which then remained fairly constant, may be interpreted as a real treatment effect. Similar to other studies [19, 20] our data shows that complete normalization of plasma TG values is rarely achieved in patients with sHTG. In patients with sHTG, those with a history of acute pancreatitis had particularly high TG values. Moreover, patients with sTG levels and pancreatitis all had co-factors known to increase the risk for pancreatitis [18], potentially suggesting that sHTG on its own will not predispose to acute pancraetitis. On the other hand, Pedersen et al. [10] recently showed that even those with mild hypertriglyseridemia (TG > 2 mmol/L) were at increased risk for acute pancreatitis, after adjustment for several known confounders.

In the subgroup of six hypertriglyceridemic patients we found that the PBMC mRNA expression of several key mediators in lipid metabolism was altered as compared to controls. Thus, the patients were characterized by higher mRNA expression of *CPT-1A*, *CPT-2*, and *LIPE*, concomitantly with lower mRNA expression of *ABCG1* compared to controls. Defects in CPT-1 and CPT-2 are associated with hypertriglyceridemia in humans [21], whereas increased CPT-1 activity is associated with reduced TG concentrations and reduced muscle- and liver steatosis in rats [22], suggesting that the high TG levels in the present study population is not caused by decreased β-oxidation. *CPT* is a PPARα target gene and, as expected, in the patients treated with fibrates, the PBMC mRNA expression of *CPT1A* and *CPT2* were higher compared to controls, thereby counteracting the increased TG levels. Hormone sensitive lipase (*LIPE*) is also a PPARα target gene, inducing hydrolyses of TG to fatty acids, and this enzyme was also upregulated in PBMC from patients. Thus, it is tempting to hypothesize that the up-regulation of these PPARα sensitive enzymes may represent counteracting mechanisms to down-regulate TG levels. In contrast to the up-regulation of genes involved in fatty acid metabolism, PBMC from these patients had decreased mRNA expression of *ABCG1*, a transporter involved in mediating cholesterol efflux, potentially leading to reduced cholesterol efflux capacity.

In the present study we combined data of prevalence and treatment with analyses to characterize the molecular etiology to strengthen the observations. We acknowledge the fact that since our pooled estimate of sHTG was based on several large surveys, we cannot conclude that this is fully representative for the general Norwegian population. Another possible weakness in large population surveys is that sick people usually participate to a lower extent than healthy. Additionally we cannot exclude possible confounding due to differences in life-styles. Since sHTG is associated with a considerable comorbidity the present data may thus have underestimated the true prevalence of sHTG in the population. On the other hand, patients with sHTG are referred to the Lipid Clinic from all over Norway increasing the representativeness for this population. A fasting TG value was not always available. Moreover, lack of a control group and a participation rate of 58% may have led to a selection bias in the 65 patients with persistent sHTG. Regarding the PBMC sub-study, a limitation is that few patients were included and only male subjects were studied.

## Conclusions

In conclusion, sHTG was present in 1/1000 subjects based on a pooled estimate from several large surveys. Furthermore sHTG was associated with serious comorbidity, in particular CVD, and was associated with an imbalance of genes involved in lipid metabolism. Notably, sHTG is easy and inexpensive to detect and deserve increased attention to limit serious organ complications.

## Additional file

Additional file 1: Table S1. Inclusion and exclusion criteria for patients with sHTG for detailed analyses. **Table S2.** List of lipid-related genes analyzed in peripheral blood mononuclear cells from patients with severe hypertriglyceridemia. **Table S3.** Baseline biochemical characteristic of 65 patients with severe hypertriglyceridemia. **Table S4.** Combinations of medications used at end of study among the 65 patients with hypertriglyceridemia. **Table S5.** Characteristics of the six patients with hypertriglyceridemia subjected to in-depth molecular analyses. (PDF 258 kb)

## Abbreviations

ABCG1: ATP-binding cassette, sub-family G, member 1
APO: Apolipoprotein
BMI: Body mass index
CONOR: Cohorts of Norway
CPT: Carnitine palmitoyltransferase
CVD: Cardiovascular disease
GPIHBP1: Glycosylphosphatidylinositol-anchored high density lipoprotein-binding protein 1
GUSβ: Glucuronidase β
LIPE: Hormone sensitive lipase
LMF1: Lipase maturation factor 1
LPL: Lipoprotein lipase
PBMC: Peripheral blood mononuclear cells
SD: Standard deviation
sHTG: Severe hypertriglyceridemia
TBP: TATA box binding protein
TG: Triglycerides
