# Supplementary material for: Severe hypertriglyceridemia in Norway: prevalence, clinical and genetic characteristics
Source: Lipids Health Dis. 2017 Jun 12;16:115. doi: 10.1186/s12944-017-0511-9 (PMC5469061; doi:10.1186/s12944-017-0511-9)
Supplement: Additional file 1: Table S1. — Inclusion and exclusion criteria for patients with sHTG for detailed analyses. Table S2. List of lipid-related genes analyzed in peripheral blood mononuclear cells from patients with severe hypertriglyceridemia. Table S3. Baseline biochemical characteristic of 65 patients with severe hypertriglyceridemia. Table S4. Combinations of medications used at end of study among the 65 patients with hypertriglyceridemia. Table S5. Characteristics of the six patients with hypertriglyceridemia subjected to in-depth molecular analyses. (PDF 258 kb) [file 12944_2017_511_MOESM1_ESM.pdf]

Table S1

Inclusion and exclusion criteria for patients with sHTG for detailed analyses.

Inclusion criteria:

1. All individuals ≥ 18 years treated for sHTG at the Lipid Clinic from January 1st 2002 to December 31st 2007 were identified by searching the medical records for the diagnoses (ICD-10 classification): pure hypertriglyceridemia (E78.1), mixed hyperlipidemia (E78.2) and hyperchylomicronemia (E78.3).
2. Fasting triglyceride concentration measured > 10 mmol/L at three different time points 2-6 months apart (n=112).
3. Sixty-five (58%) patients returned a signed informed consent and were included in the study and came for a baseline visit.
4. Expressed willingness to participate in the study by signed informed consent

Exclusion criteria:

1. Age < 18 years
2. No fasting triglyceride concentration > 10 mmol/L at three different time points 2-6 months apart
3. Not willing to participate in the study and/or not willing to sign informed consent

Specific inclusion criteria for patients with sHTG for analyses of RNA expression of lipid-related genes in peripheral blood mononuclear cells (PBMC):

1. Age ≥ 18 years
2. Fasting triglyceride concentration measured > 10 mmol/L at three different time points 2-6 months apart.
3. Meeting to routine consultation at the Lipid Clinic in the specific time period when PBMC samples were collected for this study purpose
4. Willing to sign additional informed consent for experimental genetic analysis in addition to routine genetic analysis for diagnostic purpose

Specific exclusion criteria for patients with sHTG for analyses of RNA expression of lipid-related genes in peripheral blood mononuclear cells (PBMC):

1. Age < 18 years
2. No fasting triglyceride concentration > 10 mmol/L at three different time points 2-6 months apart
3. Not willing to participate in the study or not willing to sign informed consent

Table S2

List of lipid-related genes analyzed in peripheral blood mononuclear cells from patients with severe hypertriglyceridemia

Gene Symbol	Full name	ThermoFisher's product number
ABCA1	ATP-binding cassette, sub-family A (ABC1), member 1	Hs01059118_m1
ABCG1	ATP-binding cassette, sub-family G (WHITE), member 1	Hs01555189_m1
ACOX1	acyl-Coenzyme A oxidase 1, palmitoyl	Hs00244515_m1
CD36	CD36 molecule (thrombospondin receptor)	Hs00169627_m1
CETP	cholesteryl ester transfer protein, plasma	Hs00163942_m1
CPT1A	carnitine palmitoyltransferase 1A (liver)	Hs00912681_m1
CPT2	carnitine palmitoyltransferase II	Hs00988962_m1
GPIHBP1	glycosylphosphatidylinositol anchored high density lipoprotein binding protein 1	Hs01564843_m1
LIPE	lipase, hormone-sensitive	Hs00943410_m1
LPL	lipoprotein lipase	Hs00173425_m1
NAMPT	nicotinamide phosphoribosyltransferase	Hs00237184_m1
NPC1	Niemann-Pick disease, type C1	Hs00975249_m1
NPC2	Niemann-Pick disease, type C2	Hs00197565_m1
NR1H3	nuclear receptor subfamily 1, group H, member 3 (Liver X receptor)	Hs00172885_m1
PLTP	phospholipid transfer protein	Hs01067287_m1
PPARA	peroxisome proliferator-activated receptor alpha	Hs00947538_m1
PPARD	peroxisome proliferator-activated receptor delta	Hs00602622_m1
PPARG	peroxisome proliferator-activated receptor gamma	Hs01115512_m1
PPARGC1B	peroxisome proliferator-activated receptor gamma, coactivator 1 beta	Hs00370186_m1
SCD	stearoyl-CoA desaturase (delta-9-desaturase)	Hs01682761_m1
SREBF1	sterol regulatory element binding transcription factor 1	Hs01088691_m1
UCP2	uncoupling protein 2 (mitochondrial, proton carrier)	Hs01075225_m1

The ThermoFisher’s product number (TaqMan Microfluidic Cards) refers to information concerning the probe used to detect the target gene, e.g. the specific sequence of the probe. http://www.thermofisher.com/no/en/home/brands/applied-biosystems.html. The selection of these genes was partly based on our previous studies:

Myhrstad MC. et al. Effect of the fat composition of a single high-fat meal on inflammatory markers in healthy young women.
Br J Nutr. 2011;106:1826-1835.

Telle-Hansen VH. et al. Altered expression of genes involved in lipid metabolism in obese subjects with unfavourable phenotype
Genes Nutr. 2013; 84: 425–434.

Holven KM. et al. Subjects with low plasma HDL cholesterol levels are characterized by an inflammatory and oxidative phenotype. PLoS One. 2013;8:e78241.

Narverud I. et al. Lack of effects of a single high-fat meal enriched with vegetable n-3 or a combination of vegetable and marine n-3 fatty acids on intestinal peptide release and adipokines in healthy female subjects. Front Nutr. 2016;3:38.

Leder L. et al. Effects of a healthy Nordic diet on gene expression changes in peripheral blood mononuclear cells in response to an oral glucose tolerance test in subjects with metabolic syndrome: a SYSDIET sub-study. Genes Nutr. 2016;11:3.

Table S3

Baseline biochemical characteristic of 65 patients with severe hypertriglyceridemia

Fasting serum concentration	n*	Median	95% CI	Laboratory reference value
Glucose (mmol/L)	51	5.8	(5.6–6.1)	4.0 - 6.0
HbA1c (%)	44	5.6	(5.4–5.8)	<6.1
Insulin (ρmol/L)	26	82.5	(48.0–97.0)	18 - 173
C-peptide (nmol/L)	35	1.2	(1.0–1.4)	270 - 1290
Total-cholesterol (mmol/L)	62	8.0	(6.9–9.6)	3.3-6.9 (30-49 year)
LDL-cholesterol (mmol/L)	35	2.3	(1.9–3.3)	1.9-4.8 (30-49 year)
HDL-cholesterol (mmol/L)	58	1.1	(1.0–1.2)	Women 1.0–2.6<br>Men 0.8-2.1
Triglycerides (mmol/L)	65	12.3	(10.2–15.5)	0.5-2.6
Apo A1 (g/L)	35	1.2	(1.1–1.3)	Women 1.1–2.3<br>Men 1.0–2.0 g/L
Apo B (g/L)	40	1.0	(0.9–1.1)	0.5–1.3
Lp (a) (mg/L)	18	114.0	(87.0–315.0)	< 75
TSH (mIE/L)	43	1.8	(1.3–2.2)	0.50–3.6
FT$_4$ (ρmol/L)	43	13.9	(13.0–14.8)	8.0–21.0
CRP (mg/L)	42	2.2	(1.7–2.8)	<4
ASAT (U/L)	47	35.0	(30.0–40.0)	Women 15–35 U/L<br>Men 15–45 U/L
ALAT (U/L)	52	44.0	(35.0–56.0)	Women 10–45 U/L<br>Men 10–70 U/L
CK (U/L)	37	116.0	(97.0–158.0)	Women ≥ 18 years 35-210<br>Men 18-49 years 50-400
Creatinine (μmol/L)	43	76.0	(70.0–86.0)	Women ≥15 years 45–90<br>Men ≥15 years 60–105
Pancreas amylase (U/L)	27	24.0	(19.0–29.0)	10-65

HbA1c.glycosylated hemoglobin; LDL. low density lipoprotein; HDL. high density lipoprotein; Apo A1. Apolipoprotein A1; Apo B. Apolipoprotein B; Lp(a). Apolipoprotein B; TSH. thyroidea stimulating hormone; FT$_4$. free thyroxin; CRP. C-reactive protein; ASAT. aspartate aminotranferase; ALAT. alanine amino transferase; CK. creatine kinase; CI. confidence interval.

*n < 65 due to missing values.

Table S4

Combinations of medications used at end of study among the 65 patients with hypertriglyceridemia

Combination	n*
Statin + fish oil	16
Statin + fibrate	4
Statin + ezetimibe	1
Fish oil + fibrate	3
Statin + fish oil + fibrate	11
Statin + fish oil + ezetimibe	2
Statin + fibrate + ezetimibe	1
Statin + ezetimibe + nicotinic acid	1
Fish oil + ezetimibe + resin	1
Fish oil + ezetimibe + nicotinic acid	1
Statin + fish oil + fibrate + ezetimibe	2

*At the last consultation 43 among the 65 patients used lipid-lowering drugs.

Table S5

Characteristics of the six patients with hypertriglyceridemia subjected to in-depth molecular analyses

**Patient no.**	1	2	3	4	5	6
**HbA1c (%)**	5.4	6.9	7.7	8.1	8.6	9.6
**Lp(a) (mg/L)**	ND	517	ND	ND	157	75
**History of pancreatitis**	No	No	Yes	No	No	No
**Medication**	Bezalip 400 mg x 1; Omacor 6 capsules/d (EPA+DHA 5 g/d)	Omacor 6 capsules/d (EPA+DHA 5 g/d)	Zoloft 50 mg x 1; Omacor 6 capsules/d (EPA+DHA 5 g/d)	Atorvastatin 40 mg/d; Enalapril 2.5 mg x 1; Metformin 850 mg x 1; Amaryl 1 mg x 1; Acetyl salisylicacid 75 mg x 1; Omeprazol 20 mg x 1.	Atorvastatin 40 mg x 1; Metformin 1 g x 3; Amaryl 5 mg/d; Aprovel 300 mg x 1; Acetyl salicylic acid 75 mg x 1; Amlodipin 5 mg x1.	Bezalip 400 mg x 1; Omacor 6 capsules/d (EPA+DHA 5 g/d); Metformin 1.5 g/d
**Systolic blood pressure (mmHg)**	130	120	129	144	168	150
**Diastolic blood pressure (mmHg)**	85	76	85	99	94	97
**BMI (kg/m^2)**	26.0	40.5	35.2	29.5	37.2	33.6
**Alcohol (units/week)**	1	10	0	6	3	15
**Smoking (cigarettes/d)**	0	10	20	10	0	0
**Parents cousine marriage**	Yes	No	No	No	No	No
**Apo E genotype**	E3/E3	E2/E3	E3/E3	E3/E3	E3/E4	E2/E3

ND, not determined
